# Embedding a Dinuclear Ru─Pt Photocatalyst Into Polyampholytic Graft Copolymer Matrix Enables Prolonged Hydrogen Evolution While Allowing Catalyst Repair

**DOI:** 10.1002/chem.71568

**Published:** 2026-08-14

**Authors:** Yurii Utievskyi, Alexander K. Mengele, Mariet Puthanagady, Nikita Vashistha, Linda Zedler, Christof Neumann, Marius Müßler, Andrey Turchanin, Benjamin Dietzek‐Ivanšić, Sven Rau, Felix H. Schacher

**Affiliations:** ^1^ Institute of Organic Chemistry and Macromolecular Chemistry Friedrich Schiller University Jena Jena Germany; ^2^ Institute of Inorganic Chemistry I Ulm University Ulm Germany; ^3^ Institute of Physical Chemistry Friedrich Schiller University Jena Jena Germany; ^4^ Jena Center for Soft Matter (JCSM) Friedrich Schiller University Jena Jena Germany; ^5^ Center For Energy and Environmental Chemistry (CEEC) Friedrich Schiller University Jena Jena Germany; ^6^ Leibniz Institute of Surface Engineering Leipzig Germany; ^7^ Helmholtz Institute for Polymers in Energy Applications Jena (HIPOLE Jena) Jena Germany

**Keywords:** Dinuclear photocatalyst, Graft copolymer, in situ repair, Photocatalytic hydrogen evolution, Soft matter matrix

## Abstract

There are few molecular catalysts where active repair has been demonstrated, and one prominent example is a tetrapyridophenazine‐bridged heterodinuclear Ru‐Pt hydrogen‐evolving photocatalyst, where hydrogenation of the pyrazine ring in the bridging ligand has been shown to lead to an end of photocatalytic activity. Subsequent in situ repair by persulfate enabled catalyst reactivation, but so far at the cost of decreasing catalytic activity over several repair cycles. We now show that tailored polyampholytic graft copolymers enable electrostatic immobilization of the photocatalyst in a unimolecular soft matter matrix, thereby prolonging (photo)catalytic activity due to slower photocatalyst degradation kinetics. We attribute this to a decreased propensity for hydrogenation of the pyrazine ring and, with that, slower degradation of the bridge and improved long‐term HER activity, as evidenced by a holistic spectroscopic characterization of the molecular and electronic structure of the photocatalyst. At the same time, the graft copolymer matrix does not hinder oxidative repair of the photocatalyst, thus enabling multiple repair cycles without noticeable loss of catalytic activity. We therefore regard this as a novel blueprint for a synergistic *molecule‐in‐matrix* combination for sustainable light‐driven catalysis.

## Graphical abstract

A unimolecular polyampholytic graft copolymer matrix provides a protective confinement for an electrostatically embedded tetrapyridophenazine‐bridged heterodinuclear Ru‐Pt hydrogen evolving photocatalyst. The matrix slows the degradation kinetics of the catalyst and at the same time does not hinder its repair by ammonium persulfate.

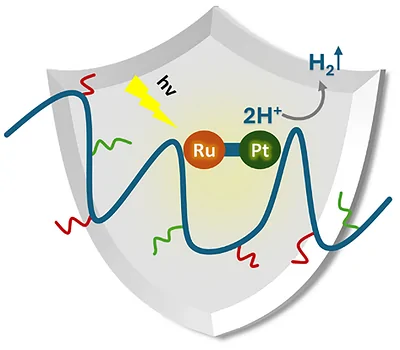

## 1 Introduction

Natural photosynthesis serves as an impressive example for the coordinated and efficient interplay between photocatalytically active centers and the surrounding soft matter matrix environment [1], enabling sustained light‐to‐energy conversion while at the same time allowing in situ repair of damaged reaction sites [2, 3, 4]. This has served as inspiration for the development of artificial photosynthesis [5], yet examples for photocatalysts capable of self‐healing [6, 7], that is the system‐immanent continuous restoration of catalytic activity without external intervention, or active repair [8, 9, 10], that is by intervention under nonoperating conditions, are still scarce [11].

One rather recent example is the heterodinuclear photocatalyst [(tbbpy)_2_Ru(tpphz)PtI_2_]^2+^ (tbbpy = 4,4´‐di‐*tert*‐butyl‐2,2´‐bipyridine, tpphz = tetrapyrido[3,2‐a:2′,3′‐c:3″″‐h:2′″,3′″‐j]phenazine), or simply Ru(tpphz)PtI_2_, which is active in the light‐driven hydrogen evolution reaction (HER). During irradiation, the activity continuously declines due to hydrogenation of the central pyrazine ring, which is complete after ca. 2 days. However, it could be shown that oxidative repair using either photochemically generated ^1^O_2_ or ammonium persulfate (APS, (NH_4_)_2_S_2_O_8_) enabled photocatalyst reactivation [9, 10]. The re‐oxidized phenazine core enables restart of catalysis and thus prolonged HER activity as well as cumulative turnover number (TON) beyond 3000 over several cycles. Nevertheless, these repair processes under harshly oxidizing conditions are likely also accompanied by side effects such as small structural alterations not changing the chromophoric properties of Ru(tpphz)PtI_2_ but still leading to a decreasing photocatalytic activity after several repair cycles and a limited overall number of attainable photocatalytic cycles.

Inspired by the matrix‐incorporation of the natural photosynthetic apparatus, our hypothesis is that the design of a suitable soft matter matrix allows for prolonged photocatalytic activity while at the same time limiting both the hydrogenation of the phenazine bridge and, if necessary, preventing side reactions during reactivation. There is a large number of reports on photocatalytic systems possessing chemically nonlinked light‐absorbers and catalysts which are incorporated into overarching matrix structures. Irrespective of the specific structure of the surrounding matrix, such as metal‐organic frameworks (MOFs) [6, 7, 12], covalent organic frameworks (COFs) [13, 14, 15], or supramolecular polymers [16, 17, 18], they typically impact the process beneficially and lead to prolonged and improved catalysis [19, 20, 21]. Such positive effects have also been already observed for heterodinuclear CO_2_‐reducing photocatalysts, leading to full substrate conversion [22, 23, 24]. Moreover, matrix‐incorporation of dinuclear metal catalysts (DMCs) proved successful for photocatalytic CO_2_ reduction [25, 26]. However, so far no studies on the effect of matrix‐incorporation of intramolecularly operating dinuclear photocatalysts for light‐driven hydrogen evolution were reported. Repair of photocatalytically active systems in biology is possible by harnessing the synergism of hierarchically structured soft matter environments. The molecular functional building blocks are embedded in rigid protein matrices, which are themselves embedded in a lipid bilayer, the thylakoid membrane. After fatal damage during photocatalysis, this protein‐protein bond is dissolved, the isolated damaged proteins are repaired, and the functional entity is reassembled within the thylakoid membrane. Such degree of synergy has, to the best of our knowledge not been observed in artificial systems.

Matrix‐integration requires balancing solubility and functionality of the underlying polymeric material to both ensure attractive interactions between host (polymer) and guest (photocatalyst) and accessibility of all reaction sites in the resulting hybrid material. Apart from negatively charged DNA as host material allowing incorporation of positively charged dinuclear complexes [27, 28, 29, 30, 31], one additional intriguing class of soft matter materials for this task are tailor‐made polyampholytic graft copolymers consisting of a polydehydroalanine (PDha) backbone and grafts of varying charge and polarity [32]. Hereby, the backbone mainly acts as anchoring site for (oppositely charged) photocatalysts due to attractive electrostatic interactions, while the side chains mediate overall solubility. Such materials have been shown to effectively disperse carbon nanotubes [33] or fragrances [34], but have also been used as matrices for light‐driven HER using [Mo_3_S_13_]^2−^ molecular catalyst (CAT) in combination with organic photosensitizers (PS) in an intermolecular photochemical mechanism [35]. Upon crosslinking, the resulting hydrogels could be employed for both HER and water oxidation catalysis (WOC) [36], while at the same time allowing for reversible pH‐dependent anchoring of catalytically active building blocks and, with that, facilitating exchange of degraded CAT or PS [37].

Hence, our approach was to design a polyampholytic graft copolymer as a unimolecular soft matter matrix for Ru(tpphz)PtI_2_ with the aim to increase its stability against bridging ligand (BL) hydrogenation while preserving photocatalytic activity and the ability for in situ repair. For this, we chose heterograft copolymers featuring a PDha backbone and both poly(ethylene glycol) (PEG—solubility) and 2‐acrylamido‐2‐methylpropane sulfonic acid (AMPS—additional sites for immobilization) side chains. This in combination with the photocatalyst resulted in photocatalytically active aggregates in solution, which exhibited a reduced rate for BL hydrogenation and, importantly, could be repaired using APS without leading to a decreased H_2_ formation in the follow‐up cycles—thus showcasing a straightforward approach to advanced *molecule‐in‐matrix* reactivity.

## 2 Results and Discussion

### 2.1 Formation of the Ru(tpphz)PtI_2_@PDha‐*g*‐AMPS‐*g*‐mPEG_1000_ Assembly

For the preparation of a unimolecular soft matter matrix for Ru(tpphz)PtI_2_, we envisioned a heterograft copolymer with two different grafted side chains based on PDha. Hereby, PEG serves to improve solubility in aqueous‐organic media independent of the pH value whereas AMPS provides additional negatively charged anchoring sites for the positively charged photocatalyst. The PDha‐*g*‐AMPS‐*g*‐mPEG_1000_ heterograft copolymer was synthesized in five steps (Figures 1 and S1). First, *tert*‐butoxycarbonylaminomethylacrylate (*t*BAMA) monomer was obtained by dehydration of *N*‐(*tert*‐butoxycarbonyl)‐D‐serine methyl ester and then polymerized *via* nitroxide‐mediated radical polymerization (NMP) in dioxane using a 400:1 monomer to initiator ratio to result in poly(*tert*‐butoxycarbonylaminomethylacrylate) (P*t*BAMA). The resulting average chain length was calculated to be 180 repeating units, based on *t*BAMA conversion determined via ^1^H NMR using the internal standard method (Figure S2). Subsequent deprotection to PDha using trifluoroacetic acid (TFA) and step‐wise grafting of AMPS in an aza‐Michael addition, followed by nucleophilic epoxide ring‐opening for PEG resulted in PDha‐*g*‐AMPS‐*g*‐mPEG_1000_ heterograft copolymer (Figures S3–S5).

**FIGURE 1:**
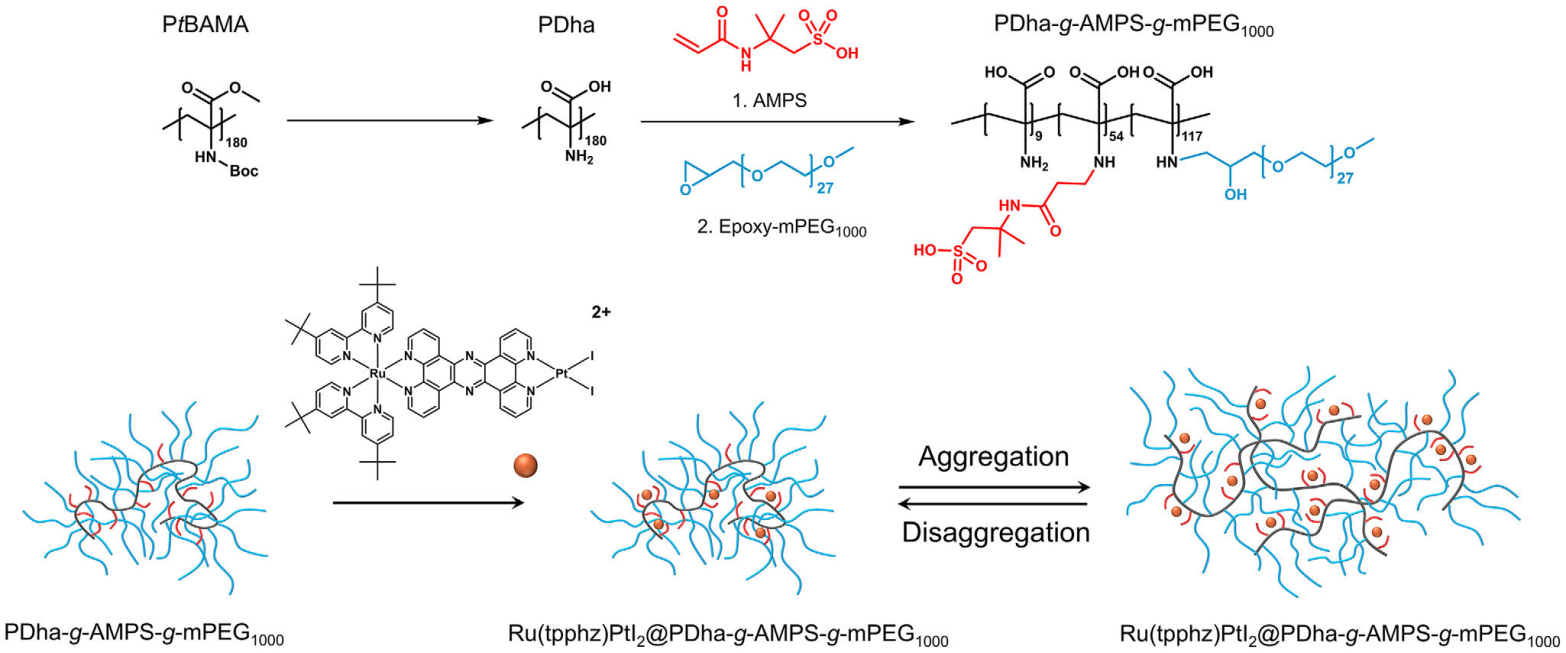
Schematic synthesis of the PDha‐*g*‐AMPS‐*g*‐mPEG_1000_ heterograft copolymer, uptake of the Ru(tpphz)PtI_2_ photocatalyst, and subsequent reversible aggregation into loose colloids in 45/30/25, H_2_O/MeCN/TEA (v/v/v).

According to ^1^H NMR, the degree of functionalization (DoF) with AMPS was around 30 % (PDha‐*g*‐AMPS, Figure S4), and further 65 % for PEG (PDha‐*g*‐AMPS‐*g*‐mPEG_1000_, Figure S5). The size‐exclusion chromatograms of P*t*BAMA, PDha‐*g*‐AMPS and PDha‐*g*‐AMPS‐*g*‐mPEG_1000_ are provided in Figure S6. The resulting heterograft copolymer exhibited solubility in aqueous media independent of the pH value. For immobilization of Ru(tpphz)PtI_2_, and the formation of photocatalyst/heterograft copolymer assembly (Ru(tpphz)PtI_2_@PDha‐*g*‐AMPS‐*g*‐mPEG_1000_), 4 mg of the heterograft copolymer were dissolved in 2 mL (2 mg∙mL^−1^) of 45/30/25, H_2_O/MeCN/TEA (v/v/v) by volume; TEA = triethylamine) containing 60 µmol∙L^−1^ of the Ru(tpphz)PtI_2_ photocatalyst. Hereby, the formation of the Ru(tpphz)PtI_2_@PDha‐*g*‐AMPS‐*g*‐mPEG_1000_ is driven by attractive electrostatic interactions between the (overall) negatively charged PDha backbone, AMPS side chains, and the positively charged photocatalyst. At the given ratio, we assume that one heterograft copolymer chain (with calculated number‐average molecular weight of 144 kg∙mol^−1^) carries on average 5 photocatalyst molecules (see also Figure 1). In an additional experiment, we varied photocatalyst/heterograft copolymer ratio at constant Ru(tpphz)PtI_2_ molarity (60 µmol∙L^−1^) and found out that a fourfold increase of the ratio leads to a slight increase of TON of Ru(tpphz)PtI_2_@PDha‐*g*‐AMPS‐*g*‐mPEG_1000_ in light‐driven HER (Table S1). This might be related to changes in the aggregation state of the photocatalyst/heterograft copolymer assemblies, and an increased tendency for secondary aggregation might be applicable at 2 mg∙mL^−1^.

Investigations of Ru(tpphz)PtI_2_@PDha‐*g*‐AMPS‐*g*‐mPEG_1000_ using dynamic light scattering (DLS) showed both the presence of heterograft copolymer unimers of approximately 6 nm (both for the pristine heterograft copolymer solutions as well after uptake of the photocatalyst) and loose colloidal aggregates of about 20 nm in diameter (Figure 2B). As the solutions are transparent and no precipitation occurs even after prolonged storage, we anticipate that both unimers and aggregates are active in light‐driven HER. This was also confirmed by transmission electron microscopy (TEM) after depositing the solutions on carbon‐coated TEM grids (Figure 2A).

**FIGURE 2:**
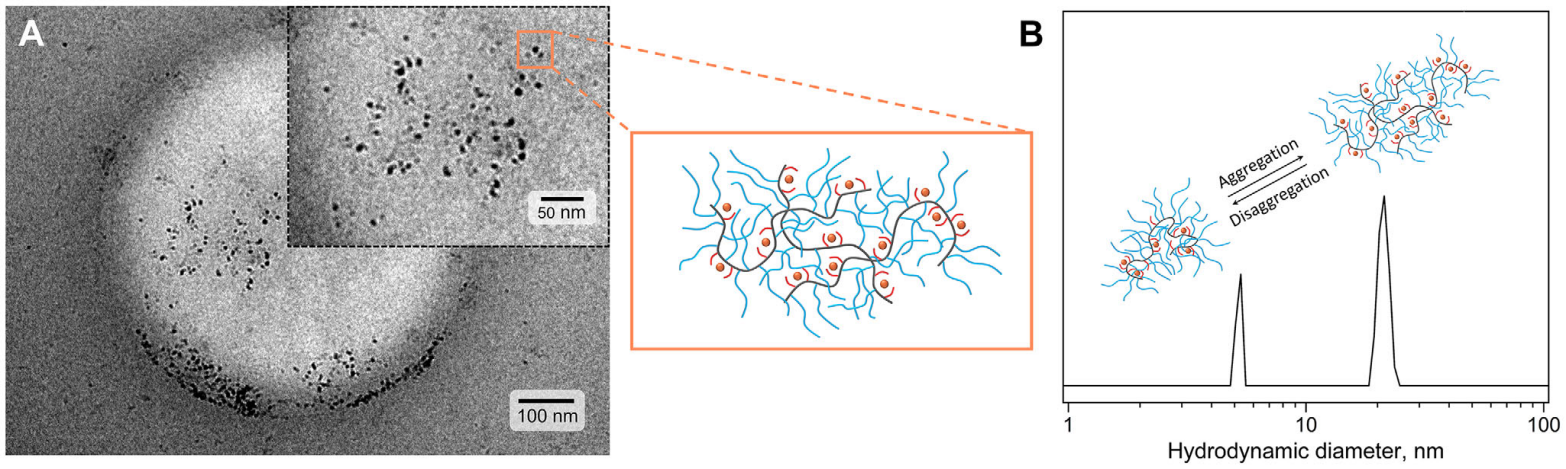
TEM micrograph (inset shows an increased magnification, A) and DLS CONTIN plot of the Ru(tpphz)PtI_2_@PDha‐*g*‐AMPS‐*g*‐mPEG_1000_ assembly (B) in H_2_O/MeCN/TEA, 45/30/25 (v/v/v) at 2 mg∙mL^−1^ heterograft copolymer and 60 µmol∙L^−1^ Ru(tpphz)PtI_2_.

### 2.2 Light‐Driven HER and In Situ Repair

Initially, we investigated the performance of the Ru(tpphz)PtI_2_@PDha‐*g*‐AMPS‐*g*‐mPEG_1000_ assembly in light‐driven HER under 460 nm blue LED irradiation in photoreactor type A (see instrumentation, Figure S15) varying H_2_O/MeCN/TEA solvent compositions to account for changes in solvation of Ru(tpphz)PtI_2_@PDha‐*g*‐AMPS‐*g*‐mPEG_1000_. Conventional photocatalysis with Ru(tpphz)PtI_2_ utilizes high amounts of MeCN, which modulates solvent environments but is a nonbenign organic solvent. Increasing the H_2_O/MeCN ratio is therefore desirable. It was varied while keeping the TEA (electron donor) volumetric share constant: 27/48/25, 35/40/25, and 45/30/25 ratios of H_2_O/MeCN/TEA (v/v/v) were used. The performance of Ru(tpphz)PtI_2_@PDha‐*g*‐AMPS‐*g*‐mPEG_1000_ was compared to the molecular photocatalyst Ru(tpphz)PtI_2_ at each solvent composition in triplicates. The amount of evolved hydrogen was quantified with headspace gas chromatography (GC) right after terminating irradiation and then converted into corresponding TONs (Tables S2–S4) [38]. It was found that the TON of the Ru(tpphz)PtI_2_@PDha‐*g*‐AMPS‐*g*‐mPEG_1000_ assembly at solvent composition of 45/30/25, H_2_O/MeCN/TEA (v/v/v) was higher than that of the molecular photocatalyst by around 45% (Table S2). Similar but less pronounced impact was observed at 35/40/25, H_2_O/MeCN/TEA (v/v/v) solvent composition (Table S3). The 45/30/25, H_2_O/MeCN/TEA (v/v/v) solvent composition was therefore chosen for further studies. The presence of the heterograft copolymer hereby allows working at increased fractions of water, due to optimized charge balance and solubilizing effect of the polymer.

To follow photocatalytic HER kinetics under these conditions, we used a 19 mL Schlenk flask equipped with a rubber septum, which enabled multiple sampling of the gas phase as previously reported [9]. HER was carried out in photoreactor type B (see instrumentation) using triplicates with reaction volume of 5 mL and extended irradiation time, the evolved hydrogen was quantified using in situ headspace GC (Figure 3 for graphs of TON and turnover frequency (TOF) versus irradiation time).

**FIGURE 3:**
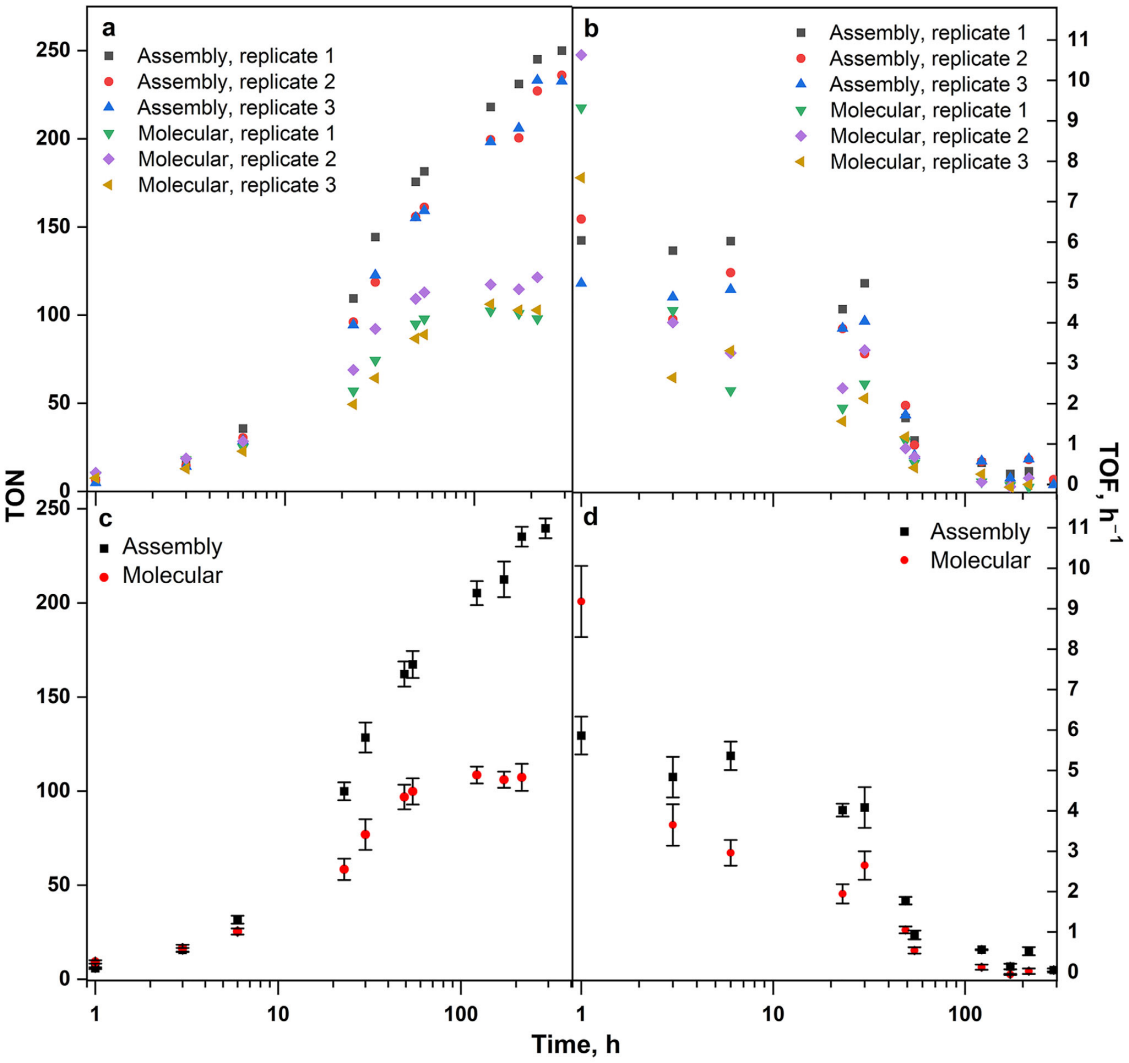
Kinetics of light‐driven HER of Ru(tpphz)PtI_2_@PDha‐*g*‐AMPS‐*g*‐mPEG_1000_ and molecular photocatalyst Ru(tpphz)PtI_2_ at 45/30/25, H_2_O/MeCN/TEA (v/v/v) solvent composition under 465 nm blue LED irradiation. (a) TON of triplicates. (b) TOF of triplicates. (c) Averaged TON (mean ± standard error). (d) Averaged TOF (mean ± standard error).

Both, the Ru(tpphz)PtI_2_@PDha‐*g*‐AMPS‐*g*‐mPEG_1000_ assembly and the molecular photocatalyst Ru(tpphz)PtI_2_ exhibited gradual TON increase during the first 50 h of irradiation, reaching values of 165 and 100, respectively (Figure 3a,c and Table S5). The TOF of both catalysts was decaying over this time period, decreasing from 6 to 0.5 h^−1^ and from 9 h^−1^ to nearly 0 h^−1^, respectively (Figure 3b,d and Table S5). Further irradiation led only to a statistically insignificant increase of Ru(tpphz)PtI_2_ TON from 100 to 110. Meanwhile, Ru(tpphz)PtI_2_@PDha‐*g*‐AMPS‐*g*‐mPEG_1000_ sustained catalytic activity for the next 150 h at TOF of around 0.5 h^−1^, reaching TON value of 240 before complete deactivation of the catalyst. Thus, incorporation of Ru(tpphz)PtI_2_ into PDha‐*g*‐AMPS‐*g*‐mPEG_1000_ heterograft copolymer resulted in a fourfold extension of catalyst lifetime, which resulted in a more than twofold increase in TON.

The induction times of Ru(tpphz)PtI_2_@PDha‐*g*‐AMPS‐*g*‐mPEG_1000_ and molecular photocatalyst Ru(tpphz)PtI_2_ were estimated in a separate one‐hour kinetic experiment under the same reaction conditions and were found to be less than 10 min (Figure S7), which aligns well with what has previously been found for the molecular photocatalyst [39]. Very recent investigations with advanced H_2_ detection devices indicate an induction phase within similar time domains [10]. According to mercury poisoning test, light‐driven HER follows exclusively a molecular mechanism in the presence of the heterograft copolymer (Table S6) [40, 41, 42, 43]. This is further supported by ‐ray photoelectron spectroscopy (XPS), showing the characteristic doublet of the Pt 4f_7/2_ and Pt 4f_5/2_ orbitals after prolonged HER at binding energies of 72.7 and 75.9 eV, respectively, which are characteristic of Pt (II) (Figure S8).

At this point, our hypothesis was that immobilization of the photocatalyst into the heterograft copolymer matrix significantly slowed down the di‐hydrogenation of the phenazine core of the BL, resulting in inactive Ru(tpphzH_2_)PtI_2_ form, although not fully suppressing this process. This could explain the extended lifetime and, as a result of this, higher observed TON of the immobilized photocatalyst.

To elucidate whether the heterograft copolymer confinement has an effect on oxidative repair of the catalyst, we performed five consecutive HER/repair cycles using the reaction conditions from Table S2 until the point where light‐driven activity significantly declined. After each HER, Ru(tpphzH_2_)PtI_2_ was oxidized in situ to Ru(tpphz)PtI_2_ using an excess of APS as described earlier (Figure 4 and Table S7) [10]. No decrease in TON after each repair was visible over five consecutive HER/repair cycles, what suggested that the heterograft copolymer confinement does not hinder the interaction between the photocatalyst and APS. We attribute slightly elevated TONs to APS traces in the mixtures, which might result in additional catalyst reactivation during HER [10].

**FIGURE 4:**
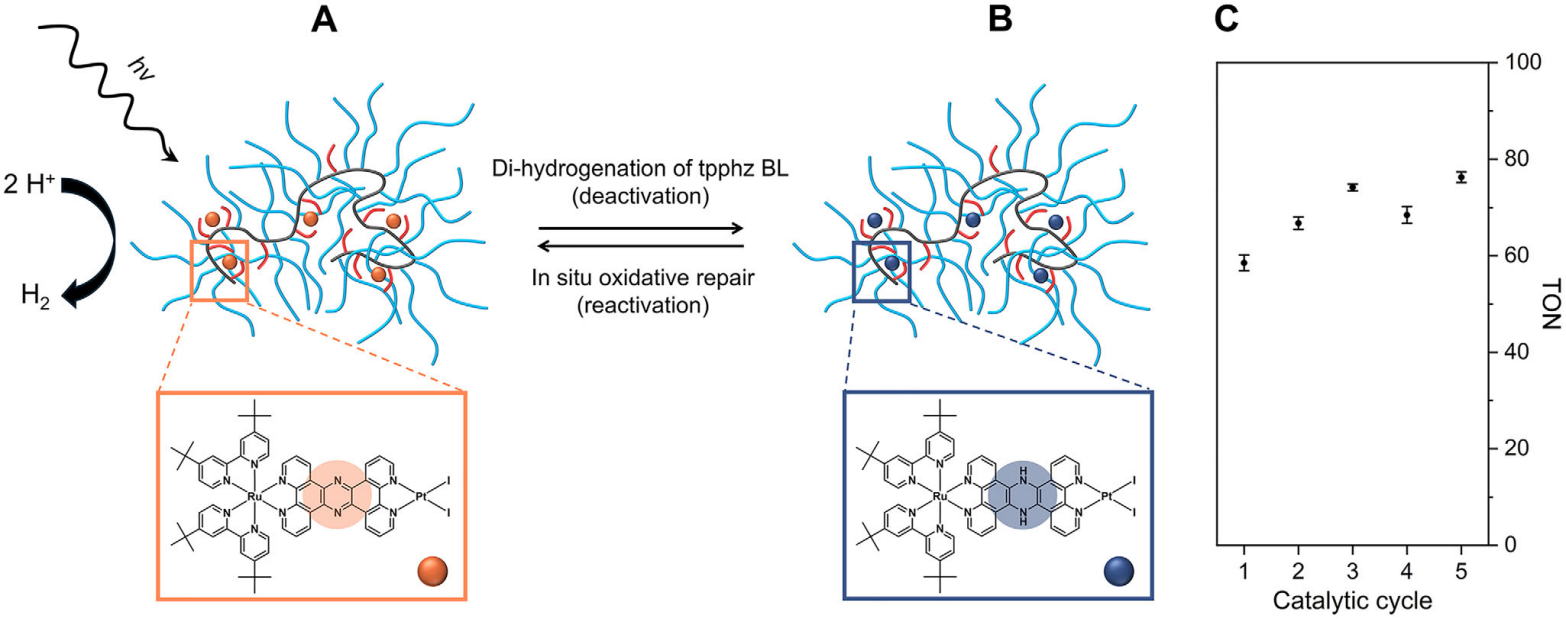
Schematic depiction of light‐driven HER using the Ru(tpphz)PtI_2_@PDha‐*g*‐AMPS‐*g*‐mPEG_1000_ assembly (A), deactivation upon di‐hydrogenation to Ru(tpphzH_2_)PtI_2_@PDha‐*g*‐AMPS‐*g*‐mPEG_1000_ (B) and reactivation by in situ oxidative repair (from B to A). (C) TON (mean ± standard error) of Ru(tpphz)PtI_2_@PDha‐*g*‐AMPS‐*g*‐mPEG_1000_ over 5 consecutive catalytic cycles at 45/30/25, H_2_O/MeCN/TEA (v/v/v) solvent composition under 460 nm blue LED irradiation. The photocatalyst was reactivated (repaired) in situ using circa 400 eq. of APS. Duration of irradiation: first cycle–66 h; second cycle–90 h; third cycle– 87 h; fourth cycle–72 h; fifth cycle–91 h.

### 2.3 Spectroscopic Characterization

The headspace GC analysis of the catalytic mixtures was complemented by extensive in situ spectroscopic measurements conducted under comparable conditions as used in light‐driven catalysis. These experiments are summarized in Figure 5: The UV‐Vis spectrum of Ru(tpphz)PtI_2_@PDha‐*g*‐AMPS‐*g*‐mPEG_1000_ undergoes a red shift under photocatalytic conditions, and a broad absorption feature emerges between 500 and 700 nm (Figures 5a1‐2). This feature is rather broad and does not allow for the discrimination between singly hydrogenated species, which have been discussed as intermediates of the photocatalytic cycle, and the doubly hydrogenated species, which constitute a deactivated species of the catalyst [44]. However, it is very apparent that the relative intensities of the MLCT absorbance at app. 450 nm and the new band of the semi‐ and fully hydrogenated tpphz‐based transition are very different for the molecular photocatalyst, nearly identical intensities from the start, whilst RuG exhibits a 550/450 nm absorbance ratio 0.65 to 1 at 1 h and 0.8 to 1 at 2 h (Figure 5a2). In the *in situ* resonance Raman experiments during catalysis, a new peak emerges at 1350 cm^−1^ associated with in plane vibrations of C─C/C─N of reduced tpphz BL (tpphzH_2_) (Figure 5b1) [45]. Together, these findings corroborate the formation of a new molecular species, the features of which are in line with the literature‐reported features of doubly hydrogenated forms of the photocatalyst [9].

**FIGURE 5:**
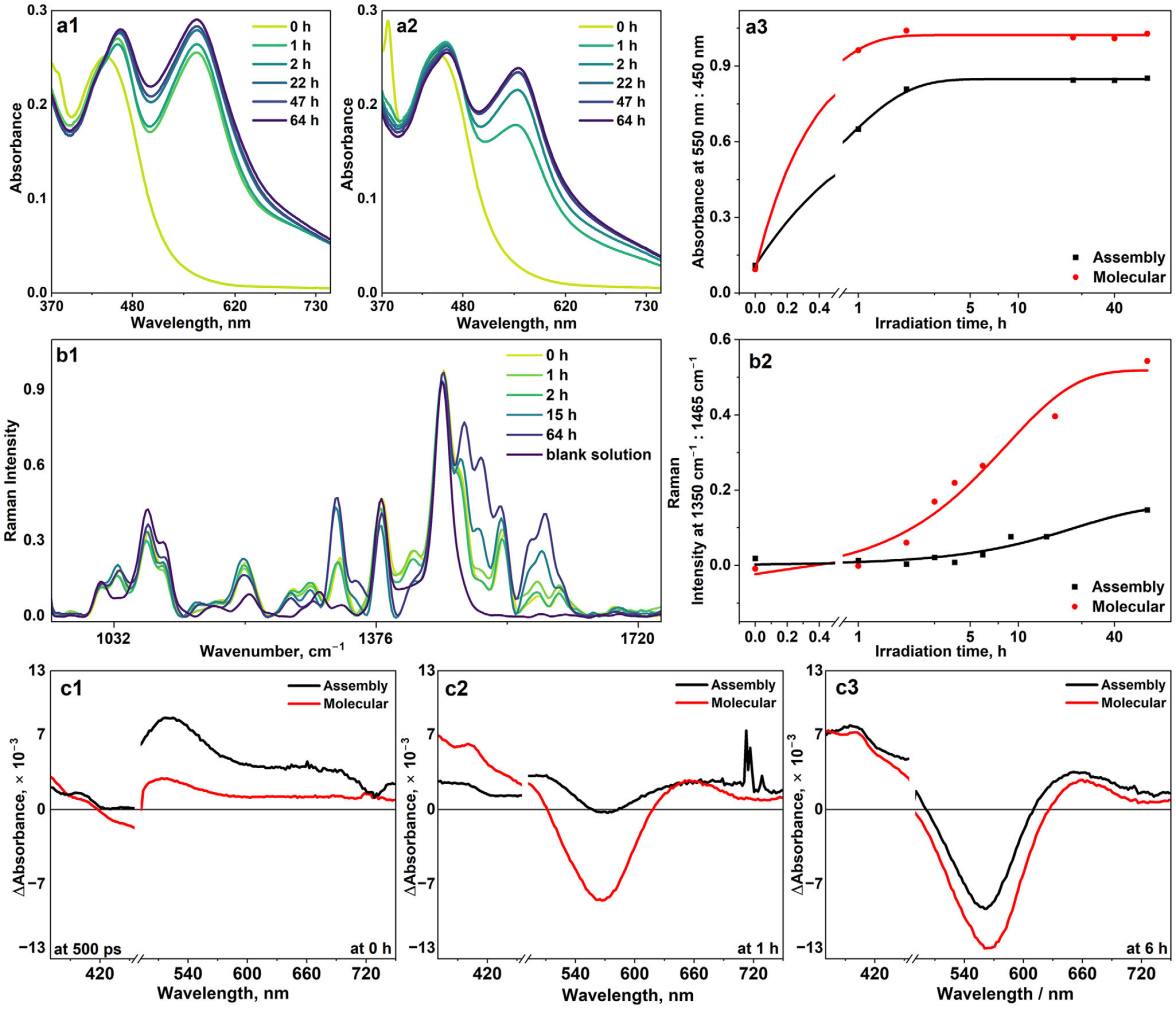
Plot of (a1) UV/Vis absorption of molecular Ru(tpphz)PtI_2_ after given irradiation time (using 460 nm blue LED with 10 – 12 mW). (a2) UV‐Vis absorption of Ru(tpphz)PtI_2_@PDha‐*g*‐AMPS‐*g*‐mPEG_1000_ after given irradiation time. (a3) Ratio of absorbance at 550 nm: 450 nm vs. irradiation time. (b1) Raman spectra after the given irradiation time of Ru(tpphz)PtI_2_@PDha‐*g*‐AMPS‐*g*‐mPEG_1000_. (b2) Raman intensity ratio of the intensity measured at 1350 cm^−1^ and 1465 cm^−1^ versus time of LED irradiation. TA spectra of molecular Ru(tpphz)PtI_2_ and Ru(tpphz)PtI_2_@PDha‐*g*‐AMPS‐*g*‐mPEG_1000_ at 500 ps at 0 h (c1), 1 h (c2), and 6 h (c3) irradiation, respectively.

However, the changes in the UV/Vis absorption spectra evolve on characteristic timescale of less than 1 h (0.4 h and 0.7 h for Ru(tpphz)PtI_2_@PDha‐*g*‐AMPS‐*g*‐mPEG_1000_ assembly and molecular Ru(tpphz)PtI_2_, respectively) (Figure 5a3), while the changes reflected in the resonance Raman data occur on a much longer timescale (23 h and 8 h for Ru(tpphz)PtI_2_@PDha‐*g*‐AMPS‐*g*‐mPEG_1000_ and molecular Ru(tpphz)PtI_2_, respectively) (Figure 5b2). Furthermore, the timescale on which the resonance Raman signals evolve matches the timescale on which the TOF of the catalyst ceases. Figure S14 shows that the temporal evolution of the TOF values aligns with the number of active photocatalysts, which is calculated under the assumption that the spectral evolution reflected in the resonance Raman signal shows the formation of degraded photocatalysts. Thus, we conclude that the temporally slow evolution of the resonance Raman signals reflects the decreased rate of degradation of the photocatalysts, while the formation of the intense UV/Vis absorption at around 550 nm indicates the formation of catalytically active intermediates.

In addition, in situ transient absorption (TA) spectroscopy highlights the changes of excited‐state features during photoredox catalysis (see supporting information for the full set of data, Figures S9–S13). The discussion here focuses on the TA features recorded at 500 ps after optical excitation at 470 nm of both systems (red—molecular Ru(tpphz)PtI_2_, black—Ru(tpphz)PtI_2_@PDha‐*g*‐AMPS‐*g*‐mPEG_1000_). For the assembly, the spectrum recorded prior to photocatalysis (at 0 h) (Figure 5c1) reflects contributions of excited state absorption (ESA) between 490 and 590 nm attributed to singly reduced, singly protonated BL (tpphzH) from previously reported literature studies [9]. During irradiation, a new ground state bleach (GSB) feature emerges between 500–650 nm (relatively intense for molecular photocatalyst), which reflects the bleach of the ligand centered transition associated with doubly hydrogenated tpphz ligand, that is the inactivation of the photocatalyst and a prolonged ESA (completely absent for molecular photocatalyst) associated with singly hydrogenated tpphz BL, that is the activation of the photocatalyst. Following the comparative time‐trace in both systems (Figures 5c1–c3) which describes the emergence of the inactivated catalyst and disappearance of the activated catalyst, we conclude that the polymer prolongs the time during which the catalyst remains active in solution. The apparent rate constants derived from decay of the Raman intensity ratio signals (1350 cm^−^^1^:1465 cm^−^^1^) are 3.3 and 1.2∙10^−^^5^ s^−^^1^ for the molecular and polymer‐embedded systems, respectively (Figure 5b2), indicating similar decay orders and faster degradation in the molecular system. As the Raman‐derived decay of the active catalyst closely parallels the trend observed in TOF decay (Figure S14), a relationship between structural degradation (hydrogenation) and catalytic deactivation can be observed. To quantify this behaviour, exponential fits to both datasets were employed which yielded comparable time constants: 8 h (Raman) and 7 h (TOF) for the molecular system, and 23 h (Raman) and 45 ± 12 h (TOF) for the polymer‐embedded system. This level of disagreement can be accounted to the differing sensitivities of Raman spectroscopy (structural changes) and TOF measurements (catalytic function). Notably, the longer time constants for the polymer‐embedded system indicate slower degradation, what is in line with the higher amount of evolved hydrogen relative to the molecular counterpart.

## 3 Conclusion

We present the first example where embedding of a photocatalyst within a heterograft copolymer, as a blueprint for a tailored soft matter matrix, leads to slower photocatalyst degradation kinetics, resulting in higher photocatalyst TON compared to the nonembedded photocatalyst. Importantly, the soft matter matrix does not impede oxidative repair of the photocatalyst, enabling multiple consecutive repair cycles without decrease in photocatalytic activity (TON). Our combined kinetic, spectroscopic, and structural data points to the formation of moderately well‐defined colloidal aggregates which are active in light‐driven HER and where the photocatalyst can be repeatedly re‐activated by oxidative repair upon hydrogenation of the tpphz BL. We were able to follow the evolution of the latter by in situ resonance Raman spectroscopy and align this data with the kinetics from light‐driven catalytic HER. The developed tracking of the photocatalytic activity with resonance Raman provided much more accurate information of the catalytic system, if compared to conventional UV/Vis spectroscopy. While surely further studies are required to quantitatively understand the effects of graft copolymer structure and composition on the catalytic activity of a given photocatalyst, we think that our study clearly shows a proof‐of‐concept for light‐driven catalysis in tailored soft matter environments. As the employed heterograft copolymer possesses several acid‐base active side chains, proton management during the catalysis and the deactivation steps might play an important role in determining the very positive effects on overall photocatalytic performance. Thinking this further, this concept can be easily extended to macroscopic materials such as hydrogels or films where even transport processes can be controlled in a better way.

## 4 Materials and Methods

### 4.1 Materials

*N*‐*tert*‐butoxycarbonyl‐D‐serine methyl ester (98%, Carbolution), 1,4‐dioxane (anhydrous, 99.8%, Sigma Aldrich), trifluoracetic acid (≥99.9%, Carl Roth), 2‐acrylamido‐2‐methyl‐1‐propansulfonic acid (99%, Sigma‐Aldrich), monomethoxy‐PEG epoxide, MW 1k (Creative PEGWorks), MeCN (HiPerSolv CHROMANORM, VWR Chemicals), triethylamine (≥99.5%, Carl Roth), ammonium persulfate (≥98.0%, Sigma‐Aldrich; APS) were received from commercial suppliers.

**Synthesis of [(tbbpy)_2_Ru(tpphz)PtI_2_](PF_6_)_2_ (Ru(tpphz)PtI_2_) and *tert*‐butoxycarbonyl‐aminomethylacrylate (*t*BAMA)**: Both Ru(tpphz)PtI_2_ and *tert*‐butoxycarbonylaminomethylacrylate (*t*BAMA) were synthesized according to previously published methodologies [34, 39].

**Synthesis of poly(*tert*‐butoxycarbonylaminomethylacrylate) (P*t*BAMA)**: P*t*BAMA was synthesized via nitroxide‐mediated radical polymerization. 2 g (10 mmol) of *t*BAMA, 9.5 mg (25 µmol) of BlockBuilder initiator, and 4.8 mg (16.5 µmol) of SG1 were dissolved in 4 mL of dioxane in the 60 mL Schlenk flask. The solution was degassed via four free‐pump‐thaw cycles and then the flask was filled with argon. The reaction was carried out at 70°C (the oil bath was preheated) for 10 min under stirring, the reaction was quenched by immersing the Schlenk flask into liquid nitrogen. The *t*BAMA conversion was estimated using trioxane as an internal standard. The product was obtained by precipitation in cold hexane. The precipitate was separated via centrifugation, washed several times with cold hexane, and dried under high vacuum. The polymer was obtained as a white powder (0.8 g, 40%).

^1^H NMR (300 MHz, CDCl_3,_ δ): 6.25‐4.75 (broad s, ─NH─) ppm; 3.70 (s, ─O─CH_3_) ppm; 3.40‐1.90 (m, ─C─CH_2_─) ppm; 1.90‐1.10 (m, ─C(O)O─C(CH_3_)_3_) ppm.

SEC (THF, PMMA calibration): M_n_ = 60 kg∙mol^−1^; M_w_ = 102 kg∙mol^−1^; Đ = 1.69.

**Synthesis of poly(dehydroalanine) PDha**: 0.8 g of P*t*BAMA was dissolved in 7.5 mL of trifluoroacetic acid (TFA) and sealed in the 20 mL microwave vial. The reaction mixture was heated at 50°C for 3 h under stirring. The product was obtained by precipitation in cold methanol. The precipitate was centrifuged, washed two times with cold methanol and afterwards dried under high vacuum. The polymer was obtained as a yellow powder (220 mg, 60%).

^1^H NMR (300 MHz, D_2_O, NaOD, δ): 3.31 (s, ─O─CH_3_) ppm; 2.97‐1.75 (m, ─C─CH_2_─) ppm.

Note: the remaining methyl ester protecting groups at 3.31 ppm are completely removed under basic conditions in the next synthetic step [32].

**Synthesis of PDha‐*g*‐AMPS**: 100 mg of PDha and 15 mL of basic aqueous solution (pH = 13.5‐14.0, KOH, adjusted with pH‐meter) containing 1,2 g (5.8 mmol) of the 2‐acrylamido‐2‐methylpropane sulfonic acid (which was neutralized) were transferred into the 20 mL microwave vial. The vial was sealed, and the solution was stirred for 10 min at room temperature to completely dissolve the polymer. Upon complete polymer dissolution, the reaction solution was heated at 65°C for 48 h under stirring. Upon reaction completion, the solution was cooled to room temperature and slowly neutralized with 1 M aqueous solution of hydrochloric acid. The solution was then dialyzed against water for 3–4 days (MWCO = 1.0 kDa) and freeze‐dried for 2 days. The graft copolymer was obtained as a white powder (140 mg).

^1^H NMR (300 MHz, D_2_O, NaOD, δ): 3.69 (dt, 6.0 Hz, 1.5 Hz, ─CH_2_─) ppm; 3.35 (s,─CH_2_─) ppm; 2.90‐1.90 (m, ─C─CH_2_─) ppm; 2.44 (t, 6.0 Hz, ─CH_2_─) ppm; 1.44 (s,─CH_3_) ppm.

SEC (H_2_O, PEG calibration): M_n_ = 101.5 kg∙mol^−1^; M_w_ = 203.5 kg∙mol^−1^; Đ = 2.0

**Synthesis of PDha‐*g*‐AMPS‐*g*‐mPEG_1000_**: 100 mg of PDha‐*g*‐AMPS graft copolymer and 15 mL of basic aqueous solution (pH = 13.5‐14.0, KOH, adjusted with pH‐meter) containing 750 mg of epoxy‐mPEG_1000_ were transferred into the 20 mL microwave vial. The vial was sealed, the mixture was stirred for 10 min at room temperature to completely dissolve the graft copolymer. The reaction solution was subsequently heated at 60°C for 72 h under stirring. Upon reaction completion, the reaction solution was cooled to room temperature and slowly neutralized with 1 M aqueous solution of hydrochloric acid. The solution was subsequently dialyzed against deionized water for 4–5 days (MWCO = 3.5 kDa) and then freeze‐dried for three days. The heterograft copolymer was obtained as a white powder (160 mg).

^1^H NMR (300 MHz, D_2_O, NaOD, δ): 3.80‐3.55 (m, ─O─CH_2_─CH_2_─) ppm; 3.37 (s, ─O─CH_3_) ppm; 2.94‐2.08 (m, ─C─CH_2_─) ppm; 1.46 (s, ─CH_3_) ppm.

SEC (H_2_O, PEG calibration): M_n_ = 82.5 kg∙mol^−1^; M_w_ = 155.5 kg∙mol^−1^; Đ = 1.9

**Reaction solution preparation for light‐driven HER**: All the catalytic reaction mixtures and stock solutions were prepared in the glovebox under inert atmosphere (argon) in the dark, using deaerated water, acetonitrile (MeCN) and triethylamine (TEA) as solvents. Fresh Ru(tpphz)PtI_2_ photocatalyst stock solution was prepared for each series of HERs in a 4 mL screw cap vial by dissolving the defined mass of [(tbbpy)_2_Ru(tpphz)PtI_2_](PF_6_)_2_ in acetonitrile. Subsequent dilution of the stock solution during reaction mixture preparation resulted in final molarity of 60 µmol∙L^−1^. As an example, the reaction stock solution of 45/30/25 – H_2_O/MeCN/TEA (v/v/v) solvent ratio was prepared by mixing 7.2 mL H_2_O, 2.4 mL MeCN, 2.4 mL of catalyst stock solution in MeCN, and 4.0 mL TEA in a 20 mL screw cap vial. The resulting stock solution was shaken vigorously and left to equilibrate for 10 min. Subsequently, 13 mg of the PDha‐*g*‐AMPS‐*g*‐mPEG_1000_ heterograft copolymer were dissolved in 6.5 mL of the reaction stock solution in a 10 mL screw cap vial, vigorously shaken and left to equilibrate for 15–20 min. In the last step, 2 mL aliquots of the stock solutions with and without the polymer were transferred to the GC‐vials, preparing in total three replicates for each catalytic system. The GC vials were sealed with crimp caps and taken out of the glovebox for light‐driven HER in photoreactor type A (see Figure S15). Reaction solutions for HER in 19 mL Schlenk flasks were prepared similarly but with different volumes, HERs in Schlenk flasks were carried in photoreactor type B (see Figure S16).

**Reactivation of the Ru(tpphz)PtI_2_ photocatalyst with ammonium persulfate (NH_4_)_2_S_2_O_8_**: The catalytic solutions with Ru(tpphz)PtI_2_@PDha‐*g*‐AMPS‐*g*‐mPEG_1000_ at 45/30/25, H_2_O/MeCN/TEA (v/v/v) solvent composition were prepared in 4 mL GC vials identically to how it was done in previous experiments. After irradiation and headspace GC measurements, the vials were brought back to the Ar‐filled glovebox, and the catalytic solutions from all vials were transferred with syringe into one 20 mL vial. The vial was then left opened for 45 min to release all generated hydrogen gas. After that, around 400 eq. of ammonium persulfate (as a solid) were added to the solution, the vial was slightly shaken and kept opened for another 40 min. The color of the solution changed back to orange, implying successful photocatalyst reoxidation. Afterwards, the 2 mL aliquots of the solution were transferred to new 4 mL GC vials for the next light‐driven HER cycle. Reactivation with APS was performed 4 times in a row.

**Size exclusion chromatography (SEC)**: SEC measurements of PDha‐*g*‐AMPS and PDha‐*g*‐AMPS‐*g*‐mPEG_1000_ graft copolymers were performed on a Jasco system (Tokyo, Japan) equipped with an AS‐2051 Plus autosampler, DG‐2080‐53 degasser, PU‐980 pump, RI‐2031 Plus RI detector, UV‐975 UV detector, PSS SUPREMA guard/1000/30 Å (10 µm particle size) column, and a Jasco oven. Aqueous 0.08 M Na_2_HPO_4_/0.05% NaN_3_ (pH = 9) solution was used as an eluent at 1 mL∙min^−1^ flow rate at 30°C. The system was calibrated using polyethylene glycol (PEG) standards (194 – 969,000 g∙mol^−1^).

SEC measurements of P*t*BAMA polymer were performed on a Shimadzu system (Kyoto, Japan) equipped with CBM‐20A controller, SIL‐10AD vp autosampler, DGU‐14A degasser, LC‐10AD vp pump, RID‐10A RI detector, SPD‐10AD vp UV detector, PSS guard/Linear M (5 µm particle size) column, and CTO‐10AC vp oven. THF was used as an eluent at 1 mL∙min^−1^ flow rate at 30°C. The system was calibrated using poly(methyl methacrylate) (PMMA) standards (410 – 981,000 g∙mol^−1^).

**Dynamic light scattering (DLS)**: DLS measurements were carried out using LS spectrometer (LS Instruments AG, Fribourg, Switzerland) equipped with a diode‐pumped solid‐state (DPSS) continuous‐wave (CW) laser emitting at 660 nm (Cobolt AB, Solna, Sweden), avalanche photodiode detector (APD), a thermostat, and goniometer which allows measurements in the angular range between 8° and 153°. Light scattering of a sample, placed in a 4 mL test tube (borosilicate glass) at 22°C, was recorded at angle of 90°. The particle size‐distribution function was obtained using LS‐spectrometer software (provided by LS Instruments AG) by applying CONTIN algorithm.

**Transmission Electron Microscopy (TEM)**: For TEM imaging, a 200 kV FEI Tecnai G2 20 (Hillboro, OR, USA) microscope equipped with a 1k×1k Olympus MegaView camera (Münster, Germany) was used, with the acceleration voltage set to 120 kV. 10 µL of the sample solution was deposited on a carbon support film on a 400‐mesh copper grid manufactured by Quantifoil Micro Tools (Großlöbichau, Germany). The films were hydrophilized in an Argon plasma produced by a Diener Electronics (Ebhausen, Germany) plasma oven for 120 s prior to sample deposition. After blotting the excess with filter paper, the samples were allowed to air dry before introduction to the microscope.

**Photoreactor type A (**Figure S15): Photocatalytic HERs, using 4 mL GC‐vials, were carried out in a custom made, modular 3D‐printed photoreactor manufactured by Professor Dirk Ziegenbalg research group (Ulm University, Ulm, Germany) [46]. The irradiation chamber of the reactor had the dimensions of 90 × 90 × 100 mm. The chamber was manufactured form polylactic acid using Fused Deposition Modeling. White filament was used because of its reflective properties. The two side walls of the irradiation chamber were made with grooves (slots) in which a sample holder can be installed. The backside wall was equipped with an opening and a cooling fan on its backside. The removable front sliding wall was equipped with an opening as well to enable airflow through the irradiation chamber. The chamber could be closed from above with a lid. GC‐vials were inserted/installed into a circular sample holder. The vials were irradiated by the light source (blue LED with λ_max_ = 460 nm, LZ4‐40B208‐0000, LED Engin Inc.) installed at the bottom of the chamber and operating at 70% of its maximum power (I = 0.7 A, U = 12.4 V). The lamp was installed onto a heat sink.

**Photoreactor type B (**Figure S16): Photocatalytic hydrogen evolution reactions (HERs)s, using 19 mL Schlenk flasks, were carried out in a custom‐made photoreactor consisting of a line of four‐walled stainless‐steel chambers, each equipped with four cooling fans and two metal clams, one at the top and one at the bottom. Schlenk flasks were positioned in the middle of each chamber using the upper metal clamp. Four in‐house‐made LED arrays with blue LED lamps (λ_max_ = 465 nm, model NSPB500AS, NICHIA Corporation, Anan, Japan), mounted onto a 3D‐printed holder (held by the bottom clamp), were positioned around each Schlenk flask.

**Quantification of Molecular Hydrogen With Headspace Gas Chromatography (GC) (Light‐driven HER in GC‐vials)**: The amount of hydrogen gas was quantified via headspace GC by taking a 100 µL aliquot from the GC‐vial's headspace with a Hamilton syringe. The aliquot was manually injected into a Shimadzu Nexis GC‐2030 gas chromatograph with helium as carrier gas, SH‐Msieve 5A column and barrier discharge ionization detector (BID). Helium flow rate was set to 2.75 mL∙min^−1^, column oven temperature was set to 70°C. The GC was calibrated by injection of different hydrogen/argon mixtures with known hydrogen amount.

**Quantification of molecular hydrogen with headspace GC (Light‐driven HER in Schlenk flasks)**: The amount of the hydrogen gas was quantified via headspace GC by taking a 100 µL aliquot from the Schlenk flask's headspace with a Hamilton syringe. The aliquot was manually injected into a Bruker Scion GC‐MS system with argon as a carrier gas, Restek ShinCarbon ST Micropacked Column (mesh 80/100, 2 m x 0.53 mm I.D.) and thermal conductivity detector. Argon flow rate was set to 30 mL∙min^−1^, column oven temperature was set to 40°C, detector temperature was set to 200°C. The GC was calibrated by mixing different volumes of pure hydrogen together with argon into a Schlenk vessel.

### 4.2 ^1^H NMR Spectroscopy

^1^H NMR spectra were recorded on a Bruker Avance I 300 MHz spectrometer

**X‐ray photoelectron spectroscopy (XPS)**: XPS measurements were performed using a K‐Alpha X‐ray Photoelectron Spectrometer System (Thermo Fisher Scientific) with a monochromatic x‐ray source (Al K_α_) with a spot diameter of 400 µm and an electron detector with an energy resolution of 0.5 eV. Charge compensation during data acquisition was realized by the internal electron flood gun of the system. The spectra were calibrated using the C 1s peak (284.6 eV) and fitted using Voigt functions after background subtraction. For sample preparation, the solutions were dropcasted onto Si/SiO_2_ wafers and dried at ambient conditions.

**In Situ spectroscopy**: For in situ spectroscopic measurements, four different catalyst solutions were prepared: Solutions of the molecular photocatalyst (Ru(tpphz)PtI_2_ = 100 µmol∙L^−1^) and the Ru(tpphzH_2_)PtI_2_@PDha‐*g*‐AMPS‐*g*‐mPEG_1000_ assembly (Ru(tpphz)PtI_2_ = 100 µmol∙L^−1^, heterograft copolymer = 3.3 mg∙mL^−1^) in 45/30/25, H_2_O/MeCN/TEA (v/v/v) solvent and in 60/40, H_2_O/MeCN (v/v) solvent for the reference (in the absence of the TEA electron donor). The solutions were prepared in the glovebox under inert atmosphere (argon) and then transferred into four gas‐tight quartz cuvettes (inside glovebox) with 1 mm optical path length. Cuvettes were irradiated by Neopixel E466616 LED array consisting of 8 blue LEDs (460 nm, 10–12 mW) followed by the UV‐Vis, Raman and TA spectroscopic measurements.

**UV‐Vis absorption spectroscopy**: UV‐Vis absorption spectra were recorded in 370–700 nm range using a JASCO spectrometer V‐530 in transmission mode. For baseline correction, blanks with solvent compositions of 45/30/25, H_2_O/MeCN/TEA (v/v/v) and 60/40, H_2_O/MeCN (v/v) were used.

**Resonance Raman spectroscopy**: Resonance Raman (rR) spectra were recorded using 473 nm diode‐pumped solid‐state lasers (HB‐Laser, Germany). Raman‐scattered light was collected with an IsoPlane 160 spectrometer (Princeton Instruments, USA), equipped with a grating of 1200 grooves∙mm^−1^ and a 30 µm entrance slit to ensure high spectral resolution. A Long‐pass optical filter (Semrock, USA) matched to the excitation wavelength was used to suppress Rayleigh scattering and allow selective detection of Raman signals. The laser power at the sample was kept at 5 mW to minimize thermal effects and avoid sample degradation. H_2_O/MeCN/TEA, 45/30/25 (v/v/v) was used as an internal standard, with its characteristic Raman band at 1465 cm^−1^ (TEA) serving to calibrate both the Raman shift axis and the relative spectral intensities. The detailed data analysis of the resonance Raman spectra was carried out in the fingerprint region ranging from 550 to 1750 cm^−^^1^. Baseline correction and fluorescence removal were carried out using the asymmetric least squares (AsLS) method implemented in Origin (OriginLab, baseline/peak analysis module). The fitting parameters were set as follows: smoothing parameter (λ) = 4, asymmetry parameter (p) = 0.001, and number of iterations = 10. A convergence tolerance of 0.001 was applied as the stopping criterion. It should be noted that the parameter scaling follows the implementation in Origin. These parameters were optimized to effectively remove the strong fluorescence background arising from the Ru‐based photosensitizer while preserving the integrity of the Raman features. Subsequently, the spectra were normalized to the TEA band at 1465 cm^−1^ to ensure comparability between measurements. Finally, the processed spectra were plotted in the same manner as presented in the publication, allowing for comparison and interpretation of spectral features. To validate data authenticity, we have included representative raw (unprocessed) Raman spectra at selected irradiation times in the Supporting Information (Figure S17).

**Transient absorption spectroscopy**: Femtosecond transient (fs‐TA) absorption spectroscopy was carried out by using an amplified Ti:Sapphire laser (Libra, Coherent Inc.) which produced ∼100 fs, 800 nm pulses at 1 kHz repetition rate. This fundamental output beam is split into two beams, a pump and probe beam using a beamsplitter. The pump pulse of 470 nm and power of 500 µJ is generated by an optical parametric amplifier (OPA) and the probe pulse that is, white light (350‐700 nm) is generated through a rotating CaF_2_ crystal. The mutual polarization of the pump and probe beam is set at a magic angle of 54.7° followed by spatial and temporal overlap on the sample. For time resolved measurements, using a translational delay stage the arrival of probe pulse on the sample is delayed with respect to the pump pulse. Spectrometer equipped with a photodiode array is used to detect the intensity changes of transmitted probe as a function of delay time (Δt) between pump and probe. The data obtained is then processed using Kimopack Python package (by doing chirp and background correction) followed by a global fit of the chirp‐corrected file.

## Conflicts of Interest

The authors declare no conflicts of interest.

## Supporting information



The authors have not cited additional references within the Supporting Information. The Supporting Information includes details on synthesis and characterization of the graft copolymer, experimental details of the hydrogen evolution reactions (HERs) tests, and tabular summaries of catalytic performance under varying conditions, including catalyst repair, XPS data, TA spectroscopy data, resonance Raman data as well as photographs of the utilized photoreactors.
**Supporting File 1**: chem71568‐sup‐0001‐SuppMat.docx.

## Data Availability

The data that support the findings of this study are available from the corresponding author upon reasonable request.
