# Supplementary material for: Embedding a Dinuclear Ru─Pt Photocatalyst Into Polyampholytic Graft Copolymer Matrix Enables Prolonged Hydrogen Evolution While Allowing Catalyst Repair
Source: Chemistry. 2026 Aug 14;32(40):e71568. doi: 10.1002/chem.71568 (PMC13716008; doi:10.1002/chem.71568)
Supplement: Supplementary file 1 [file CHEM-32-e71568-s001.docx]

**Embedding a Dinuclear Ru-Pt Photocatalyst into Polyampholytic Graft Copolymer Matrix Enables Prolonged Hydrogen Evolution**

**while Allowing Catalyst Repair**

**Supporting Information**

Yurii Utievskyi,^1^ Alexander K. Mengele,^2^ Mariet Puthanagady,^3^ Nikita Vashistha,^3^ Linda Zedler,^3^ Christof Neumann,^3^ Marius Müßler,^2^ Andrey Turchanin,^3,4,5^ Benjamin Dietzek-Ivanšić,^3,6*^ Sven Rau,^2*^ Felix H. Schacher^1,4,5,7*^

1. Institute of Organic Chemistry and Macromolecular Chemistry, Friedrich Schiller University Jena, Humboldtstraße 10, 07743 Jena, Germany
2. Institute of Inorganic Chemistry I, Ulm University, Albert-Einstein-Allee 11, 89081 Ulm, Germany
3. Institute of Physical Chemistry, Friedrich Schiller University Jena, Helmholtzweg 4, 07743 Jena, Germany
4. Jena Center for Soft Matter (JCSM), Friedrich Schiller University Jena, Philosophenweg 7, 07743 Jena, Germany
5. Center for Energy and Environmental Chemistry (CEEC), Friedrich Schiller University Jena, Philosophenweg 7a, 07743 Jena, Germany
6. Leibniz Institute of Surface Engineering, Permoserstraße 15, 04318 Leipzig, Germany
7. Helmholtz Institute for Polymers in Energy Applications Jena (HIPOLE Jena), Lessingstraße 12-14, 07743 Jena, Germany

Corresponding authors: [benjamin.dietzek-ivansic@iom-leipzig.de](mailto:benjamin.dietzek-ivansic@iom-leipzig.de); [sven.rau@uni-ulm.de](mailto:sven.rau@uni-ulm.de); [felix.schacher@uni-jena.de](mailto:felix.schacher@uni-jena.de);

**Content**

| Synthesis and characterization of PDha-*g*-AMPS-*g*-mPEG_1000_………………………………………………… | 3 |
| --- | --- |
| Light-driven hydrogen evolution……………………………………………………………………………………………….. | 9 |
| Induction time…………………………………………………………………………………………………………………… | 12 |
| Mercury poisoning test and X-ray photoelectron spectroscopic analysis……………………………… | 13 |
| *In-situ* catalyst repair………………………………………………………………………………………………………………….. | 15 |
| *In-situ* spectroscopy …………………………………………………………………………………………………………………… | 16 |
| Photoreactors……………………………………………………………………………………………………………………………… | 21 |
| Raw resonance Raman spectra…………………………………………………………………………………………. | 22 |
| References………………………………………………………………………………………………………………………… | 23 |

**Synthesis and characterization of PDha-*g*-AMPS-*g*-mPEG_1000_**

**Figure S1:** Five-step synthetic route for the synthesis of PDha-g-AMSP-g-mPEG_1000_.

**Figure S2:** Estimation of the tBAMA monomer conversion during NMP via ^1^H NMR spectroscopy (in CDCl_3_), using trioxane as internal standard.

**Figure S3:** ^1^H NMR spectrum of PDha recorded in D_2_O (+2 drops of 30 % aqueous KOD per 1 mL of polymer solution in D_2_O).

**Figure S4:** ^1^H NMR spectrum of PDha-*g*-AMPS graft copolymer recorded in D_2_O (+2 drops of 30 % aqueous KOD per 1 mL of polymer solution in D_2_O)

Evaluation of the degree of functionalization (DoF) with AMPS side chains:

$$2x+2y=9.00$$

$$6y=6$$

Where $x$ is number of moles of PDha monomer units, both functionalized and non-functionalized.

$y$ is number of moles of PDha monomer units functionalized with AMPS side chain, set as 1 mol.

Consequently, x = 3.50, y = 1.

$$DoF with AMPS, \% = \frac{y}{x}\times100\%= \frac{1}{3.50}\times100\%\approx30\%$$

**Figure S5:** ^1^H NMR spectrum of PDha-g-AMPS-g-mPEG_1000_ heterograft copolymer synthesized via sequential grafting of PDha-g-AMPS graft with epoxy-mPEG_1000_. The spectrum was recorded in D_2_O (+2 drops of 30 % aqueous KOD per 1 mL of polymer solution in D_2_O).

Evaluation of the DoF with AMPS and mPEG_1000_ side chains:

$$4z+3y = 11.79$$

$$2x+2y+2z =13.05$$

$$6y =6$$

Where $x$ is number of moles of PDha monomer units, both functionalized and non-functionalized.

$y$ is number of moles of PDha monomer units functionalized with AMPS side chain, set as 1 mol.

$z$ is number of moles of PDha monomer units functionalized with mPEG_1000_ side chain.

Consequently, x = 3.33, y = 1, z = 2.20.

$$DoF with AMPS,\%=\frac{y}{x}\times100\%= \frac{1}{3.33}\times100\%\approx30\%$$

$$DoF with mPEG1000,\% = \frac{z}{x} \times100\%= \frac{2.20}{3.33} \times100\% \approx65\%$$

**Figure S6:** Size exclusion chromatograms of PtBAMA (THF, PMMA calibration) on the left and of PDha-g-AMPS and PDha-g-AMPS-g-mPEG_1000_ (H_2_O, PEG calibration) on the right.

The difference in the SEC elution volumes of PDha-*g*-AMPS and PDha-*g*-AMPS-*g*-mPEG_1000_ reflects the change in the hydrodynamic radius, which, besides molecular weight of the polymers, also strongly depends on the charge density of the polymer chains and charge screening effects. Therefore, the observed decrease in hydrodynamic radius after grafting of mPEG_1000_ chains should not be necessarily interpreted as decrease in molecular weight of the polymer, but rather as a change in the coiling of the polymer chains. Successful grafting of mPEG_1000_ chains onto PDha-*g*-AMPS was confirmed by ^1^H NMR spectroscopy after dialysis and solubility of the heterograft copolymer in water in the entire pH range and in H_2_O/MeCN/TEA (v/v/v) ternary solvent mixtures used for photocatalytic HER.

For the estimation of the number-average molecular weight (M_n_), we relied on the estimated via ^1^H NMR spectroscopy monomer conversion and DoP (Figure S2), and DoF of PDha backbone by AMPS and mPEG_1000_ side chains (Figures S4 and S5). The calculated M_n_ for PDha-*g*-AMPS and PDha-*g*-AMPS-*g*-mPEG_1000_ were 27.2 kg∙mol^−1^ and 144 kg∙mol^−1^, respectively.

**Light-driven hydrogen evolution**

Photocatalytic HER in 4 mL GC vials (2 mL reaction scale) was carried out in photoreactor type A (Tables S1-4; see also Fig. S15). Kinetics of photocatalytic HERs was measured in 19 mL Schlenk flasks (5 mL reaction scale) in photoreactor type B (Table S5; see also Fig. S16). The concentration of Ru(tpphz)PtI_2_ was 60 µmol∙L^−1^ and the heterograft copolymer concentration was varied from 0.5 to 2 mg∙mL^−1^ (Table S1). The concentrations of Ru(tpphz)PtI_2_ and heterograft copolymer were kept 60 µmol∙L^−1^ and 2 mg∙mL^−1^ for experiments from Tables S2-4. The evolved hydrogen was measured with headspace GC after irradiation termination for experiments in Tables S1-S4 and *in situ* for HER kinetics, Table S5.

**Table S1:** Photocatalytic hydrogen evolving activity (expressed in TON) of the Ru(tpphz)PtI_2_@PDha-*g*-AMPS-*g*-mPEG_1000_ assembly at different heterograft copolymer concentrations at 45/30/25, H_2_O/MeCN/TEA (v/v/v) solvent composition.

| Heterograft copolymer concentration, mg∙mL^-1^ | Replicate | Irradiation time, h | TON |
| --- | --- | --- | --- |
| 2 | 1 | 65 | 66 |
|  | 2 |  | 62 |
| 1 | 1 |  | 66 |
|  | 2 |  | 72 |
| 0.5 | 1 |  | 73 |
|  | 2 |  | 81 |

**Table S2:** Photocatalytic hydrogen evolving activity of Ru(tpphz)PtI_2_@PDha-*g*-AMPS-*g*-mPEG_1000_ assembly and molecular photocatalyst Ru(tpphz)PtI_2_ at 45/30/25, H_2_O/MeCN/TEA (v/v/v) solvent composition.

| Catalyst | Replicate | Irradiation time, h | TON |
| --- | --- | --- | --- |
| Molecular | 1 | 87 | 42 |
|  | 2 |  | 43 |
|  | 3 |  | 47 |
| Assembly | 1 |  | 64 |
|  | 2 |  | 63 |
|  | 3 |  | 63 |

**Table S3:** Photocatalytic hydrogen evolving activity of Ru(tpphz)PtI_2_@PDha-*g*-AMPS-*g*-mPEG_1000_ assembly and molecular photocatalyst Ru(tpphz)PtI_2_ at 35/40/25, H_2_O/MeCN/TEA (v/v/v) solvent composition.

| Catalyst | Replicate | Irradiation time, h | TON |
| --- | --- | --- | --- |
| Molecular | 1 | 93 | 73 |
|  | 2 |  | 68 |
|  | 3 | 117 | 64 |
| Assembly | 1 | 93 | 84 |
|  | 2 |  | 84 |
|  | 3 | 117 | 80 |

**Table S4:** Photocatalytic hydrogen evolving activity of Ru(tpphz)PtI_2_@PDha-*g*-AMPS-*g*-mPEG_1000_ assembly and molecular photocatalyst Ru(tpphz)PtI_2_ at 27/48/25, H_2_O/MeCN/TEA (v/v/v) solvent composition.

| Catalyst | Replicate | Irradiation time, h | TON |
| --- | --- | --- | --- |
| Molecular | 1 | 68 | 78 |
|  | 2 |  | 78 |
|  | 3 |  | 73 |
| Assembly | 1 |  | 57 |
|  | 2 |  | 70 |
|  | 3 |  | 68 |

**Table S5:** Kinetics of photocatalytic HER with Ru(tpphz)PtI_2_@PDha-g-AMPS-g-mPEG_1000_ assembly and molecular photocatalyst Ru(tpphz)PtI_2_ at 45/30/25, H_2_O/MeCN/TEA (v/v/v) solvent composition.

|  | Assembly | | | Molecular | | |
| --- | --- | --- | --- | --- | --- | --- |
|  | Replicate 1 | Replicate 2 | Replicate 3 | Replicate 1 | Replicate 2 | Replicate 3 |
| Time, h | Turnover number (TON) | | | | | |
| 0 | - | - | - | - | - | - |
| 1 | 6.04 | 6.57 | 4.98 | 9.32 | 10.63 | 7.59 |
| 3 | 17.62 | 14.74 | 14.25 | 17.93 | 18.65 | 12.87 |
| 6 | 35.69 | 30.46 | 28.72 | 24.9 | 28.41 | 22.79 |
| 23 | 109.44 | 96.04 | 94.39 | 56.98 | 68.9 | 49.37 |
| 30 | 144.3 | 118.68 | 122.66 | 74.4 | 92.16 | 64.28 |
| 49 | 175.61 | 155.83 | 155.3 | 94.94 | 109.14 | 86.68 |
| 54.5 | 181.6 | 161.18 | 159.26 | 97.86 | 112.9 | 88.96 |
| 122 | 218.04 | 199.5 | 198.31 | 102.28 | 117.33 | 106.2 |
| 172 | 231.13 | 200.51 | 206.01 | 100.91 | 114.64 | 102.74 |
| 215 | 245.11 | 227.11 | 233.22 | 97.92 | 121.46 | 102.77 |
| 289.5 | 249.99 | 236.03 | 232.79 |  |  |  |
| Time, h | Turnover frequency (TOF), h^−1^ | | | | | |
| 1 | 6.040 | 6.570 | 4.980 | 9.320 | 10.630 | 7.590 |
| 3 | 5.790 | 4.085 | 4.635 | 4.305 | 4.010 | 2.640 |
| 6 | 6.023 | 5.240 | 4.823 | 2.323 | 3.253 | 3.307 |
| 23 | 4.338 | 3.858 | 3.863 | 1.887 | 2.382 | 1.564 |
| 30 | 4.980 | 3.234 | 4.039 | 2.489 | 3.323 | 2.130 |
| 49 | 1.648 | 1.955 | 1.718 | 1.081 | 0.894 | 1.179 |
| 54.5 | 1.089 | 0.973 | 0.720 | 0.531 | 0.684 | 0.415 |
| 122 | 0.540 | 0.568 | 0.579 | 0.065 | 0.066 | 0.255 |
| 172 | 0.262 | 0.020 | 0.154 | −0.027 | −0.054 | −0.069 |
| 215 | 0.325 | 0.619 | 0.633 | −0.070 | 0.159 | 0.00070 |
| 289.5 | 0.066 | 0.120 | −0.006 |  |  |  |

**Induction time**

**Figure S7:** Induction time measurements. One-hour kinetics of photocatalytic HER of Ru(tpphz)PtI_2_@PDha-g-AMPS-g-mPEG_1000_ assembly and molecular Ru(tpphz)PtI_2_. Reaction setup and conditions were identical to those from Table S5).

**Mercury poisoning test and X-ray photoelectron spectroscopic analysis**

According to previous reports on the hydrogen production using binuclear photocatalysts containing Pt(II) catalytic centres, the HER might proceed via both molecular and heterogeneous mechanisms. The latter mechanism becomes the major when quantitative catalytic metal centre reduction takes place. The quantitative reduction of metal centres leads to the formation of metal nanoparticles (NPs), which can be catalytically active in HER and therefore contribute to the total TON.^[1–4]^

Mercury test was applied to rule out the potential contribution of Pt NPs to the overall hydrogen yield for the photocatalyst taken up by the copolymer and molecular photocatalyst. In the given approach, the HER is carried out in the presence of excessive amount of elemental mercury Hg(0) in the solution. In case of Pt-NPs-driven HER, the formation of the corresponding HgPt amalgam would have supressed the observed TON.^[1]^

The mercury test was carried out using two replicates with photocatalyst assembled with the copolymer containing elemental mercury (5 µL) and three replicates with molecular photocatalyst Ru(tpphz)PtI_2_, in both cases 45/30/25, H_2_O/MeCN/TEA (v/v/v) solvent composition was used. As can be seen from Table S6, no changes in TON upon elemental mercury addition were observed, what confirmed that the mechanism of the light-driven HER remains molecular for photocatalyst both in Ru(tpphzH_2_)PtI_2_@PDha-*g*-AMPS-*g*-mPEG_1000_ and molecular Ru(tpphz)PtI_2_.

To complement the results obtained from the mercury test, the samples from Table S5 were analysed via XPS. The XP spectra showed the characteristic doublet of the Pt 4f_7/2_ and Pt 4f_5/2_ orbitals after prolonged HER at binding energies of 72.7 eV and 75.9 eV, respectively, which are characteristic of Pt(II).

**Table S6:** Photocatalytic hydrogen evolving activity of Ru(tpphz)PtI_2_@PDha-*g*-AMPS-*g*-mPEG_1000_ assembly in the presence of elemental mercury (Hg, 5 µL) and of molecular catalyst in the absence of Hg. Experimental conditions and photoreactor setup were identical to that in Table S2.

| Catalyst | Replicate | Irradiation time, h | TON |
| --- | --- | --- | --- |
| Assembly + Hg | 1 | 86 | 66 |
|  | 2 |  | 62 |
| Molecular without Hg | 1 |  | 41 |
|  | 2 |  | 48 |
|  | 3 |  | 42 |

**Figure S8:** High-resolution Pt 4f XP spectra of the molecular Ru(tpphz)PtI_2_ and Ru(tpphz)PtI_2_@PDha-*g*-AMPS-*g*-mPEG_1000_ assembly after HER (samples from Table S5). The observed binding energies of 72.7 eV (Pt 4f_7/2_) and 75.9 eV (Pt 4f_5/2_) correspond to Pt(II) oxidation state.

***In-situ* catalyst repair**

**Table S7:** Active repair of Ru(tpphz)PtI_2_@PDha-*g*-AMPS-*g*-mPEG_1000_ assembly with *circa* 400 eq. of ammonium persulfate (APS). HER conditions and setup were identical to those from Table S2.

| Catalytic cycle | Replicate | | | | Irradiation time, h |
| --- | --- | --- | --- | --- | --- |
|  | 1 | 2 | 3 | 4 |  |
|  | Turnover number (TON) | | | |  |
| 1 | 60 | 55 | 53 | 60 | 66 |
| 2 (1^st^ repair) | 64 | 69 | 67 | - | 90 |
| 3 (2^nd^ repair) | 75 | 73 | 74 | - | 87 |
| 4 (3^rd^ repair) | 70 | 65 | 70 | - | 72 |
| 5 (4^th^ repair) | 77 | 75 | - | - | 91 |

***In-situ* spectroscopy**

**Figure S9:** TA spectra of Ru(tpphz)PtI_2_@PDha-*g*-AMPS-*g*-mPEG_1000_ in 45/30/25, H_2_O/MeCN/TEA (v/v/v) after the indicated irradiation time (a-f; 0 h - 64 h) for delay time ranging from 0.5 ps to 7000 ps (λ_ex_ = 470 nm with 500 µW power).

**Figure S10:** TA spectra of molecular photocatalyst Ru(tpphz)PtI_2_ in 45/30/25, H_2_O/MeCN/TEA (v/v/v) after the indicated irradiation time (a-f; 0 h - 64 h) for delay time ranging from 0.5 ps to 7000 ps (λ_ex_ = 470 nm with 500 µW power).

**Figure S11:** TA spectra of molecular photocatalyst in 60/40, H_2_O/MeCN (v/v) after the given irradiation time (a-f; 0 h - 64 h) for delay time ranging from 0.5 ps to 7000 ps (λ_ex_ = 470 nm with 500 µW power).

**Figure S12:** TA spectra of Ru(tpphz)PtI_2_@PDha-*g*-AMPS-*g*-mPEG_1000_ in 60/40, H_2_O/MeCN (v/v) after the given irradiation time (a-f; 0 h – 64 h) for delay time ranging from 0.5 ps to 7000 ps (λ_ex_ = 470 nm with 500 µW power).

**Figure S13:** Flow chart showing the experimental procedure for in situ optical spectroscopy.

For optical *in situ* spectroscopy, once the sample was prepared UV-Vis and resonance Raman spectra were recorded followed by transient UV-Vis absorption (TA) measurement. For TA measurements, after excitation of sample by a pump laser of 470 nm, the probe pulse monitors changes in the excited state at different delay times over a range of 0.5 ps - 7 ns which is controlled by the movement of a translational delay stage. A single scan (shots 600) gives information of the excited state behavior at all interested delay times. To obtain a better signal to noise ratio, multiple scans are taken and an averaged spectrum is obtained which takes approximately 30-40 min depending on the parameters selected. This is followed by steady-state UV-Vis and resonance Raman measurements again after TA measurement to follow sample’s degradation by laser pulse, if any. The sample is then irradiated using an LED source for an hour, and the same measurements (UV, resonance Raman (both before and after TA) and TA) were recorded. Similarly, the process was continued for cumulative irradiation times of 1 h, 2 h, 17 h, 40 h and 64 h.

**Figure S14:** Combined plots of TOF vs irradiation time (Right y-axis) and X vs irradiation time (Left y-axis), where X equals maximum of the corresponding Raman intensity ratio curve (Figure b2) minus interpolated value at the time point. The plot at the top depicts the X(t) and TOF(t) functions for both molecular photocatalyst and the assembly. The separate bottom plots show X(t) and TOF(t) functions scaled in their min-max range for clearer illustration of the correlation between these functions for both molecular photocatalyst and the assembly.

**Photoreactors**

**Figure S15:** Photograph of photoreactor type A.

**Figure S16:** Photograph of photoreactor type B.

**Raw resonance Raman spectra**

**Figure S17:** Raw resonance Raman spectra (prior to any background subtraction or processing) after given irradiation times of Ru(tpphz)PtI_2_@PDha-g-AMPS-g-mPEG_1000_ (in H_2_O/MeCN/TEA, 45/30/25 (v/v/v)) for the illustration of the strong fluorescence background originating from the Ru-based photosensitizer.

**References**

[1]	M. Lämmle, A. K. Mengele, G. E. Shillito, S. Kupfer, S. Rau “Stability of Catalytic Centres in Light‐Driven Hydrogen Evolution by Di‐ and Oligonuclear Photocatalysts” *Chem. – Eur. J.* **2023**, *29*, e202202722.

[2]	M. Lämmle, T. D. Pilz, R. J. Kutta, M. Müßler, A. K. Mengele, H. Görls, F. W. Heinemann, S. Rau “Insights into the different mechanistic stages of light-induced hydrogen evolution of a 5,5′-bisphenanthroline linked RuPt complex” *Dalton Trans.* **2022**, *51*, 15282–15291.

[3]	M. G. Pfeffer, B. Schäfer, G. Smolentsev, J. Uhlig, E. Nazarenko, J. Guthmuller, C. Kuhnt, M. Wächtler, B. Dietzek, V. Sundström, S. Rau “Palladium versus Platinum: The Metal in the Catalytic Center of a Molecular Photocatalyst Determines the Mechanism of the Hydrogen Production with Visible Light” *Angew. Chem. Int. Ed.* **2015**, *54*, 5044–5048.

[4]	P. Lei, M. Hedlund, R. Lomoth, H. Rensmo, O. Johansson, L. Hammarström “The Role of Colloid Formation in the Photoinduced H_2_ Production with a Ru^II^−Pd^II^ Supramolecular Complex:  A Study by GC, XPS, and TEM” *J. Am. Chem. Soc.* **2008**, *130*, 26–27.
